# Access to oral health care and its social determinants across the lifespan in the United States

**DOI:** 10.3389/froh.2025.1619983

**Published:** 2025-09-11

**Authors:** Yau-Hua Yu

**Affiliations:** Department of Periodontology, Tufts University School of Dental Medicine, Boston, MA, United States

**Keywords:** access to care, oral health, social determinants of health, quality of life, questionnaire, lifespan, *All of Us* research program

## Abstract

**Background:**

Disparities in healthcare access, driven by socioeconomic status and social determinants of health (SDOH), contribute to poor health outcomes. While prior studies established the relationship between SDOH and care access, fewer have explored their joint relationships with social satisfaction and health challenges across the lifespan. Rather than assessing direct associations between dental care utilization and physical or mental difficulties, this study examines broader interrelationships among SDOH, access to oral health care, and self-reported health challenges.

**Methods:**

A cross-sectional study using a lifespan approach–by examining participants within discrete age groups–was conducted on 127,886 individuals aged 18 years and older who participated in the *All of Us* research program and completed the “Basics”, “Overall Health” and “Health Care Access and Utilization” questionnaires. The distribution of participants' SDOH and self-reported health difficulties was presented and stratified by dental care utilization, income group and age across the lifespan. Multivariate logistic regression analyses were performed to assess the associations between SDOH and access to oral health care.

**Results:**

Across age groups, a consistent trend of disadvantaged social determinants associated with lacking oral health care utilization was noted. Young participants (18–35 years old) were the most likely to report not having received oral health care within the past 12 months (32.2%), worse mental health (29.6%, fair/poor), emotional problems (31.8%), and difficulties in concentrating or remembering (18%). Notably, young adults who did not visit a dentist within 12 months were also more likely to report not visiting a medical doctor (18.1%), being unable to afford copayment (69%), and more frequently using emergency or urgent care (20.2%). No insurance coverage [odds ratio (OR) = 1.67, 95% confidence interval (CI): 1.52–1.84], annual income less than $35,000 (OR = 3.79, 95% CI: 3.58–4.01), and housing instability (OR = 1.38, 95% CI: 1.32–1.44) were all significantly associated with lack of dental care.

**Conclusion:**

This study confirms that SDOH—particularly income and housing instability—significantly impact individuals' ability to afford and access healthcare services, including dental care. These disparities were most pronounced among the youngest age group. Our findings support future policy interventions aimed at integrating dental care into overall healthcare, especially during early adulthood.

## Introduction

Oral health is a critical component of overall health and well-being (1, 2), yet it remains one of the most inequitable areas within healthcare (3, 4). Despite advances in preventive services and treatment modalities, large segments of the U.S. population—particularly those from lower-income backgrounds, racially marginalized communities (5), and individuals with disabilities—continue to face persistent barriers to accessing routine dental care (6, 7).

While growing evidence highlights the role of social determinants of health (SDOH) in shaping access to oral health care, less is known about how psychosocial well-being and functional health challenges vary across age groups and intersect with oral health care utilization. This study aims to examine differences of these physical and mental challenges across age groups to contextualize disparities in oral health care access. Although cross-sectional, the analysis adopts a lifespan-oriented approach—comparing oral health care utilization across distinct age strata—a strategy frequently used in public health to examine disparities at different life stages.

Most oral health disparities research has focused on specific age groups (such as children or older adults) (8–10), particular vulnerable populations (e.g., pregnant women and people with disabilities) (11), or traditional socioeconomic variables such as income, insurance status, or healthcare workforce distributions (12). Such studies often give less attention to how dental care access interplays with physical function, mental health, and subjective social satisfaction (13, 14). This leaves a critical gap in the life course-oriented and intersectional understanding of oral health inequities.

The *All of Us* Research Program, developed by the United States National Institute of Health, provides a uniquely diverse and representative dataset for examining these disparities. Its large-scale design and breath of demographic, health, and access-related variables allow for stratified analysis across the lifespan (15). In this study, the *All of Us* dataset was leveraged to examine the prevalence and predictors of dental care utilization across age groups, incorporating social and structural determinants as well as self-reported physical limitations and mental health conditions.

Rather than treating mental or physical health as outcomes, we include them as contextual features to better understand the broader landscape of unmet oral health needs. This multilevel perspective is guided by frameworks such as the social ecological model (SEM) (16) and the World Health Organization's (WHO) social determinants of health (SDOH) framework (17), both of which emphasize how individual, interpersonal, and structural factors—like income, insurance, housing, and disability—shape health care access across the lifespan.

Ultimately, we aim to clarify how disparities in dental care access relate to broader social and health vulnerabilities. Special attention is given to young adults, whose high mental and emotional burden underscores the urgency of integrated, equity-driven approaches to oral and overall health.

## Materials and methods

### Description of the *All of Us* research program

The United States *All of Us* research program aims to engage a cohort of one million or more US participants, with a focus on including populations that have historically been under-represented in biomedical research. Adults 18 years and older who have the capacity to consent and reside in the USA or a US territory at present are eligible. Several recruitment methods were available: direct enrollment through the *All of Us* website; invitation from partner healthcare providers, such as hospitals and community health centers; and enrollment at outreach events where individuals could learn about the program. Details of the *All of Us* cohort have been described previously (15). The *All of Us* research program collects health-related data and makes them broadly available for research uses (18). Health data are obtained through the electronic medical record and through participant surveys. Survey templates can be found on our public website: https://www.researchallofus.org/data-tools/survey-explorer/. *All of Us* does not use a statistical survey sampling framework for obtaining a nationally representative sample. The *All of Us* curated data repository version 8 (CDR v8), registered tier access, was used in this report and its last update was in February 2025. The cutoff enrollment date of participant data was from May 8th, 2018, to October 1st, 2023.

### Ethics statement

Informed consent for all participants is obtained in person or through eConsent. The protocol was reviewed by the Institutional Review Board (IRB) of the *All of Us* Research Program. The *All of Us* IRB follows the regulations and guidance of the NIH Office for Human Research Protections for all studies, ensuring that the rights and welfare of research participants are overseen and protected uniformly (19). This project was conducted in the *All of Us* Research Workbench cloud computing environment using the de-identified curated data (CDR version 8, February 2025). Results reported are in compliance with the *All of Us* Data and Statistics Dissemination Policy disallowing disclosure of group counts under 20.

### Sample size finalization

To investigate dental care access, the analytical sample was derived from participants who completed the *All of Us* “Health Care Access and Utilization” survey (*N* = 305,860). Participants with missing data on age, gender, or race were excluded, resulting in 297,959 individuals eligible for further merging with the “Basics” and “Overall Health” survey responses. Following this merge, individuals with missing values in any of the study covariates were removed. The final analytical sample consisted of 127,886 participants. A detailed summary of missing data and the sample attrition is provided in Supplementary Figure S1. Additional comparisons of various *All of Us* study populations based on the questionnaires are shown in the Box accompanying Supplementary Figure S1.

### Outcome variables from the health care access and utilization questionnaires

Oral health care utilization was assessed based on the participants' answer to the question: “During the past 12 months, have you seen or talked to a dentist or orthodontist? (Yes/No)”. While this serves as a proxy indicator for access, utilization may not reflect unmet need for instances where care was sought but not received. Participants who skipped this question or answered “Don't Know” are considered as missing values. For comparative understanding, the outcome of “cannot afford copay” is included in this study, which is determined by the participants' answer to the question: “If you get sick or have an accident, how worried are you that you will be able to pay your medical bills? (Not at all vs. Somewhat worried/Very worried)”. Similarly, participants who skipped this question or answered “Don't Know” are considered as missing values. This variable was used to reflect financial strain, and while not a measure of denied or forgone care, it provides insight into perceived cost-related barriers that may influence care-seeking behavior. The detail information of these survey questions can be searched in the *All of Us* Research Hub Data Browser (https://databrowser.researchallofus.org/survey/health-care-access-and-utilization).

### Co-variates for social determinants of health (SDOH)

Guided by SEM (16), variables were included across multiple levels: individual level, age, gender, race, nativity (US-born or not), education, income, self-reported physical, mental and cognitive difficulties; interpersonal level, marital status and social satisfaction; organizational level, usual source of care (e.g., ER vs. doctor's office), whether insurance is accepted, employment status, and time since last medical visit; community level, housing conditions (own/rent/other), duration in residence, and housing stability concern; and policy level, insurance status, affordability of care, and structural exclusion from systems of care (inferred through care utilization, financial burden, and perceived accessibility). Using the WHO SDOH framework (17), these variables are also categorized as structural (education, income, race, nativity, employment) or as intermediary (housing, functional status, psychological health, and health system access).

The SDOH covariates are derived from the “Basics” and the “Health Care Access and Utilization” survey data. In the Basics survey, individuals typically took 10–15 min to answer the basic background information such as “In what country were you born?” (USA/other), race and ethnicity (grouped into non-Hispanic white, non-Hispanic black, Hispanic, Asian and others), biological sex assigned at birth (male/female), “What is the highest grade or year of school you completed?” (grouped into high school or less, some college, and Bachelor's degree or above), “What is your current marital status?” (grouped into married, never married, or other), employment status, and the annual household income (grouped into 34,999 or less, 35,000–74,999, 75,000–149,999, 150,000 or more). Further questions related to health insurance (“Are you covered by health insurance or some other kind of health care plan?”), physical or cognitive limitations (“Because of a physical, mental, or emotional condition, do you have serious difficulty concentrating, remembering or making decisions? Yes/No”, “Do you have serious difficulty walking or climbing stairs? Yes/No”, “Do you have difficulty dressing or bathing? Yes/No”, “Because of a physical, mental, or emotional condition, do you have difficulty doing errands alone such as visiting doctor's office or shopping? Yes/No”) and housing conditions (“Do you own or tent the place where you live? Own/Rent/Other arrangement”, “How many years have you lived at your current address? Responses grouped into Less than 2 years/3–10 years/11 years or more”, “In the past 6 months, have you been worried or concerned about NOT having a place to live? Yes/No”).

### Co-variates for health care access and utilization

In addition to the outcome variables of interest, information on where participants seek care (e.g., emergency room/ER vs. doctor's office), whether their insurance is accepted, time since their last medical visit and affordability of care were retrieved from the “Health Care Access and Utilization” survey. The questions stems are: “During the past 12 months, were you told by a health care provider or doctor's office that they did not accept your health care coverage?”, “Is there a place that you USUALLY go to when you are sick or need advice about your health?”, “What kind of place do you go to most often?”, “During the past 12 months, have you seen or talked to a general doctor who treats a variety of illnesses (a physician in general practice, primary care, family medicine, or internal medicine)?”.

### Co-variates for self-reported mental health, social satisfaction and emotional problems

In the “Overall Health” survey data, participants' self-reported physical limitation, satisfaction with social activities and relationships, and general mental or emotional problems were derived from the following questions: “In general, how would you rate your mental health, including your mood and your ability to think? (Excellent/Very Good/Good/Fair/Poor)”, “In general, how would you rate your satisfaction with your social activities and relationships? (Excellent/Very Good/Good/Fair/Poor)”, “To what extent are you able to carry out your everyday physical activities such as walking, climbing stairs, carrying groceries, or moving a chair? (Completely/Mostly/Moderately/A Little/Not At All)”, “In the past 7 days, how often have you been bothered by emotional problems such as feeling anxious, depressed or irritable? (Never/Rarely/Sometimes/Often/Always)”.

### Description of the *All of Us* research workbench cloud computing environment

The *All of Us* Research Workbench is a cloud-based platform designed for researchers to access and analyze the curated datasets. Registration and access are available for those under a Data Use and Registration Agreement (DURA). All research activity takes place within a workspace, designated for individual projects, within the cloud-based platform. Various analysis tools are built-in the cloud and the analysis environment can be initiated, executed and billed within each workspace. All researchers receive a starting credit of $300 USD for usage of cloud computing environment. Details of the cost for computation is provided in the *All of Us* website (https://support.researchallofus.org/hc/en-us/articles/360039539411-Getting-Started-and-What-to-Know-About-Costs).

### Computation, R software, and statistical analysis

In this study, the RStudio (R version 4.4.0) analysis environment was created for all processes related to data extraction and data analyses using the “stats” package. Characteristics of *All of Us* participants were summarized in means and standard deviations (SD) (continuous variables) or counts and percentages (categorical variables). Distributions of demographics, social determinants and self-reported health conditions were characterized by the participants' age group (Tables 1, 2) and income group (Table 3). Data was further stratified by participants' last dental visit status (Supplementary Tables S1–S3).

**Table 1:** Characteristics of analyzed *All of Us* participants based on their age groups.

Age Groups^a^	Total	18–35	36–45	46–55	56–65	66+	***P*** ^b^
N (%)	127,886	16,832 (13.2)	20,344 (15.9)	20,532 (16.0)	25,279 (19.8)	44,899 (35.1)	
Demographics							
Age	56.39 (16.2)	29.77 (3.8)	40.63 (2.9)	50.64 (2.9)	60.66 (2.9)	73.75 (5.4)	<.001
Male	42,997 (33.6)	3,986 (23.7)	5,394 (26.5)	5,575 (27.2)	8,193 (32.4)	19,849 (44.2)	<.001
Race							<.001
Non-Hispanic White	95,034 (74.3)	10,103 (60.0)	13,828 (68.0)	14,098 (68.7)	18,780 (74.3)	38,225 (85.1)	
Non-Hispanic Black	8,637 (6.8)	1,068 (6.3)	1,368 (6.7)	1,800 (8.8)	2,145 (8.5)	2,256 (5.0)	
Hispanic	12,965 (10.1)	3,038 (18.0)	2,766 (13.6)	2,615 (12.7)	2,403 (9.5)	2,143 (4.8)	
Asian & Other	11,250 (8.8)	2,623 (15.6)	2,382 (11.7)	2,019 (9.8)	1,951 (7.7)	2,275 (5.1)	
US Born	1,15,150 (90)	15,007 (89.2)	17,956 (88.3)	17,991 (87.6)	22,522 (89.1)	41,674 (92.8)	<.001
Socioeconomics							
Education							<.001
≤High school	82,927 (64.8)	2,144 (12.7)	1,879 (9.2)	2,251 (11.0)	2,989 (11.8)	3,952 (8.8)	
Some college	31,744 (24.8)	4,594 (27.3)	4,666 (22.9)	5,251 (25.6)	6,807 (26.9)	10,426 (23.2)	
≥Bachelor's	13,215 (10.3)	10,094 (60.0)	13,799 (67.8)	13,030 (63.5)	15,483 (61.2)	30,521 (68.0)	
Marital status							<.001
Married	76,313 (59.7)	4,892 (29.1)	12,761 (62.7)	13,158 (64.1)	16,001 (63.3)	29,501 (65.7)	
Never married	25,967 (20.3)	11,337 (67.4)	5,168 (25.4)	3,181 (15.5)	3,054 (12.1)	3,227 (7.2)	
Other	25,606 (20.0)	603 (3.6)	2,415 (11.9)	4,193 (20.4)	6,224 (24.6)	12,171 (27.1)	
Income							<.001
<34,999	26,524 (20.7)	5,649 (33.6)	3,814 (18.7)	4,076 (19.9)	5,231 (20.7)	7,754 (17.3)	
35,000–74,999	32,572 (25.5)	5,297 (31.5)	5,215 (25.6)	4,170 (20.3)	5,225 (20.7)	12,665 (28.2)	
75,000–149,999	41,308 (32.3)	4,001 (23.8)	6,646 (32.7)	6,552 (31.9)	8,095 (32.0)	16,014 (35.7)	
150,000 more	27,482 (21.5)	1,885 (11.2)	4,669 (23.0)	5,734 (27.9)	6,728 (26.6)	8,466 (18.9)	
Employed	70,969 (55.5)	11,165 (66.3)	15,891 (78.1)	15,600 (76.0)	16,653 (65.9)	11,660 (26.0)	<.001
Insurance and Healthcare Access							
Insurance not accepted	15,529 (12.1)	3,242 (19.3)	3,127 (15.4)	2,907 (14.2)	3,005 (11.9)	3,248 (7.2)	<.001
Where to seek care							<.001
Doctor's office	1,16,103 (91)	13,965 (83.0)	17,631 (86.7)	18,461 (89.9)	23,530 (93.1)	42,516 (94.7)	
ER, Urgent care, etc.	11,783 (9.2)	2,867 (17.0)	2,713 (13.3)	2,071 (10.1)	1,749 (6.9)	2,383 (5.3)	<.001
Medical visit (12 mo.)	1,15,462 (90)	14,363 (85.3)	17,611 (86.6)	18,349 (89.4)	23,048 (91.2)	42,091 (93.7)	<.001
Dental visit (12 mo.)	97,763 (76.5)	11,414 (67.8)	14,502 (71.3)	15,140 (73.7)	19,212 (76.0)	37,495 (83.5)	<.001
Cannot afford copay	59,792 (15.3)	10,710 (63.6)	11,723 (57.6)	11,370 (55.4)	12,328 (48.8)	13,661 (30.4)	<.001
Housing							
Home							<.001
Owned	85,168 (66.6)	4,286 (25.5)	11,345 (55.8)	13,630 (66.4)	18,640 (73.7)	37,267 (83.0)	
Rent or Other	42,718 (33.4)	12,546 (74.5)	8,999 (44.2)	6,902 (33.6)	6,639 (26.3)	7,632 (17.0)	
How long live here							<.001
<=2 years	35,212 (27.5)	10,553 (62.7)	8,260 (40.6)	5,142 (25.0)	5,031 (19.9)	6,226 (13.9)	
3–10 years	42,574 (33.3)	4,288 (25.5)	9,853 (48.4)	8,883 (43.3)	7,868 (31.1)	11,682 (26.0)	
11+ years	50,100 (39.2)	1,991 (11.8)	2,231 (11.0)	6,507 (31.7)	12,380 (49.0)	26,991 (60.1)	
Stability concern	12,341 (9.7)	2,521 (15.0)	2,639 (13.0)	2,662 (13.0)	2,647 (10.5)	1,872 (4.2)	<.001

a Data are presented as mean (standard deviation) for continuous variables; and *n* (%) for categorical variables.

b *P*-values are from chi-square test for categorical variables or *t*-test or ANOVA test for continuous variables comparing groups. mo., months.

**Table 2:** Distribution of self-assessed physical difficulties and mental health conditions by age groups.

Age Groups^a^	Total	18–35	36–45	46–55	56–65	66+	***P*** ^b^
*N*	127,886	16,832 (13)	20,344 (16)	20,532 (16)	25,279 (20)	448,99 (35)	
Difficulties *in*							
Concentrating/remembering	14,320 (11)	3,032 (18.0)	3,154 (15.5)	2,930 (14.3)	2,858 (11.3)	2,346 (5.2)	<.001
Walking/climbing stairs	13,745 (11)	641 (3.8)	1,163 (5.7)	2,237 (10.9)	3,620 (14.3)	6,084 (13.6)	<.001
Dressing/bathing	4,289 (3.4)	302 (1.8)	527 (2.6)	915 (4.5)	1,199 (4.7)	1,346 (3.0)	<.001
Doing errands alone	8,323 (6.5)	1,314 (7.8)	1,485 (7.3)	1,743 (8.5)	1,890 (7.5)	1,891 (4.2)	<.001
Ability to complete everyday activities							<.001
Completely	89,291 (70)	13,024 (77.4)	14,692 (72.2)	13,475 (65.6)	16,438 (65.0)	31,572 (70.3)	
Mostly	19,699 (15)	2,154 (12.8)	2,940 (14.5)	3,288 (16.0)	3,946 (15.6)	7,371 (16.4)	
Moderately- Not at all	18,986 (15)	1,654 (9.8)	2,712 (13.3)	3,769 (18.4)	4,895 (19.4)	5,956 (13.3)	
Social & Emotional							
General mental health							<.001
Excellent	23,045 (18)	1,541 (9.2)	2,048 (10.1)	2,442 (11.9)	4,707 (18.6)	12,307 (27.4)	
Very Good	47,443 (37)	4,419 (26.3)	6,324 (31.1)	7,089 (34.5)	9,614 (38.0)	19,997 (44.5)	
Good	36,573 (29)	5,884 (35.0)	7,253 (35.7)	6,871 (33.5)	7,146 (28.3)	9,419 (21.0)	
Fair/Poor	20,825 (16)	4,988 (29.6)	4,719 (23.2)	4,130 (20.1)	3,812 (15.1)	3,176 (7.1)	
Social activities satisfaction							<.001
Excellent	22,744 (18)	2,543 (15.1)	2,726 (13.4)	2,777 (13.5)	4,289 (17.0)	10,409 (23.2)	
Very Good	46,403 (36)	5,639 (33.5)	6,774 (33.3)	6,832 (33.3)	8,877 (35.1)	18,281 (40.7)	
Good	35,668 (28)	5,117 (30.4)	6,342 (31.2)	6,255 (30.5)	7,095 (28.1)	10,859 (24.2)	
Fair/Poor	23,701 (18)	3,533 (21.0)	4,502 (22.1)	4,668 (22.7)	5,018 (19.9)	5,350 (11.9)	
Bothered by emotional problems							<.001
Never	24,639 (19)	1,377 (8.2)	2,040 (10.0)	2,704 (13.2)	4,891 (19.3)	13,627 (30.4)	
Rarely	41,842 (33)	3,901 (23.2)	5,818 (28.6)	6,333 (30.8)	8,560 (33.9)	17,230 (38.4)	
Sometimes	40,233 (31)	6,207 (36.9)	7,434 (36.5)	7,272 (35.4)	8,089 (32.0)	11,231 (25.0)	
Often/Always	21,172 (17)	5,347 (31.8)	5,052 (24.8)	4,223 (20.6)	3,739 (14.8)	2,811 (6.3)	

a Data are presented as mean (standard deviation) for continuous variables; and *n* (%) for categorical variables.

b *P*-values are from chi-square test for categorical variables or *t*-test or ANOVA test for continuous variables comparing groups.

**Table 3:** Characteristics of analyzed *All of Us* participants based on their income groups.

Income Groups^a^	Total	<35,000	35k-74,999	75k-149,999	150,000 +	***P*** ^b^
*N* (%)	127,886	26,524 (20.7)	32,572 (25.5)	41,308 (32.3)	41,308 (32.3)	
Demographics						
Age	56.39 (16.2)	53.18 (17.2)	56.43 (17.3)	58.03 (15.7)	56.99 (14.3)	<.001
Age group						<.001
18–35	16,832 (13)	5,649 (21.3)	5,297 (16.3)	4,001 (9.7)	1,885 (6.9)	
36–45	20,344 (16)	3,814 (14.4)	5,215 (16.0)	6,646 (16.1)	4,669 (17.0)	
46–55	20,532 (16)	4,076 (15.4)	4,170 (12.8)	6,552 (15.9)	5,734 (20.9)	
56–65	25,279 (20)	5,231 (19.7)	5,225 (16.0)	8,095 (19.6)	6,728 (24.5)	
66+	44,899 (35)	7,754 (29.2)	12,665 (38.9)	16,014 (38.8)	8,466 (30.8)	
Male	42,997 (33.6)	7,090 (26.7)	9,975 (30.6)	14,762 (35.7)	11,170 (40.6)	<.001
Race						<.001
Non-Hispanic White	95,034 (74.3)	15,126 (57.0)	23,835 (73.2)	33,450 (81.0)	22,623 (82.3)	
Non-Hispanic Black	8,637 (6.8)	3,744 (14.1)	2,459 (7.5)	1,768 (4.3)	666 (2.4)	
Hispanic	12,965 (10.1)	4,800 (18.1)	3,623 (11.1)	2,965 (7.2)	1,577 (5.7)	
Asian & Other	11,250 (8.8)	2,854 (10.8)	2,655 (8.2)	3,125 (7.6)	2,616 (9.5)	
US Born	1,15,150 (90)	23,079 (87.0)	29,731 (91.3)	37,953 (91.9)	24,387 (88.7)	<.001
Socioeconomics						
Education						<.001
≤High school	82,927 (64.8)	7,313 (27.6)	3,441 (10.6)	1,930 (4.7)	531 (1.9)	
Some college	31,744 (24.8)	10,364 (39.1)	10,490 (32.2)	8,363 (20.2)	2,527 (9.2)	
≥Bachelor's	13,215 (10.3)	8,847 (33.4)	18,641 (57.2)	31,015 (75.1)	24,424 (88.9)	
Marital status						<.001
Married	76,313 (59.7)	6,101 (23.0)	16,050 (49.3)	30,316 (73.4)	23,846 (86.8)	
Never married	25,967 (20.3)	10,379 (39.1)	8,293 (25.5)	5,356 (13.0)	1,939 (7.1)	
Other	25,606 (20.0)	10,044 (37.9)	8,229 (25.3)	5,636 (13.6)	1,697 (6.2)	
Employed	70,969 (55.5)	8,976 (33.8)	17,473 (53.6)	24,946 (60.4)	19,574 (71.2)	<.001
Insurance and Healthcare Access						
Insurance not accepted	15,529 (12.1)	4,977 (18.8)	3,941 (12.1)	3,895 (9.4)	2,716 (9.9)	<.001
Where to seek care						<.001
Doctor's office	1,16,103 (91)	23,164 (87.3)	29,472 (90.5)	38,047 (92.1)	25,420 (92.5)	
ER, Urgent care, etc.	11,783 (9.2)	3,360 (12.7)	3,100 (9.5)	3,261 (7.9)	2,062 (7.5)	
Medical visit (12 mo.)	1,15,462 (90)	23,976 (90.4)	29,412 (90.3)	37,293 (90.3)	24,781 (90.2)	0.85
Dental visit (12 mo.)	97,763 (76.5)	14,641 (55.2)	23,767 (73.0)	34,768 (84.2)	24,587 (89.5)	<.001
Cannot afford copay	59,792 (15.3)	15,778 (59.5)	18,469 (56.7)	17,952 (43.5)	7,593 (27.6)	<.001
Housing						
Home						<.001
Owned	85,168 (66.6)	7,996 (30.1)	19,855 (61.0)	33,138 (80.2)	24,179 (88.0)	
Rent or Other	42,718 (33.4)	18,528 (69.9)	12,717 (39.0)	8,170 (19.8)	3,303 (12.0)	
How long live here						<.001
< = 2 years	35,212 (27.5)	10,094 (38.1)	9,840 (30.2)	9,380 (22.7)	5,898 (21.5)	
3–10 years	42,574 (33.3)	9,180 (34.6)	10,194 (31.3)	13,574 (32.9)	9,626 (35.0)	
11+ years	50,100 (39.2)	7,250 (27.3)	12,538 (38.5)	18,354 (44.4)	11,958 (43.5)	
Stability concern	12,341 (9.7)	6,965 (26.3)	3,486 (10.7)	1,525 (3.7)	365 (1.3)	<.001

a Data are presented as mean (standard deviation) for continuous variables; and *n* (%) for categorical variables.

b *P*-values are from chi-square test for categorical variables or *t*-test or ANOVA test for continuous variables comparing groups. mo., months.

For group comparisons, chi-square test for categorical variables or *t*-test or ANOVA test for continuous variables were conducted. To comprehensively evaluate the associations between social determinants and oral health care utilization, as well as the ability to afford copay, multivariate logistic regression models were used. All covariates were entered simultaneously using the glm() function in R “stats” package, with reference groups specified in Table 4. Covariates were selected based on their relevance to the WHO SDOH framework (17), and the social ecological model (16), representing structural (e.g., income, education, employment) and intermediary determinants. Covariates in the model included age (groups), sex assigned at birth, racial ethnicity, employment, educational attainment, annual household income, marital status, insurance, and housing living conditions. The model outputs the probability/odds ratio of a binary outcome (yes/no dental care) based on multiple predictor variables (social determinants in Table 4). The model also derived 95% confidence intervals (CI) and *p*-values using the glm() function.

**Table 4:** Associations of social determinants with oral health care utilization and perceived copay affordability (two separate logistic regression models).

Variables	No Dental Visit (12 months)		Cannot Afford Copay	
	OR (95% CI)	Pv	OR (95% CI)	Pv
Intercept	0.06 (0.05–0.06)	<.000	0.27 (0.26–0.28)	<.000
Demographics				
Age (Ref: 66+ years old)				
18–35 years old	1.55 (1.46–1.64)	1.139 × 10^−52^	2.27 (2.16–2.39)	7.621 × 10^−224^
36–45 years old	1.75 (1.66–1.84)	3.319 × 10^−108^	2.06 (1.98–2.15)	1.202 × 10^−238^
46–55 years old	1.61 (1.53–1.69)	1.353 × 10^−85^	2.07 (1.99–2.16)	1.007 × 10^−272^
56–65 years old	1.46 (1.4–1.52)	5.245 × 10^−66^	1.74 (1.68–1.81)	1.656 × 10^−199^
Male (Ref: Female)	1.25 (1.21–1.29)	1.242 × 10^−46^	0.58 (0.57–0.6)	<.000
Race (Ref: Non-Hispanic White)				
Non-Hispanic Black	1.29 (1.23–1.36)	3.251 × 10^−22^	0.72 (0.69–0.76)	4.306 × 10^−38^
Hispanic	1.21 (1.16–1.27)	4.915 × 10^−16^	1.1 (1.05–1.14)	5.769 × 10^−05^
Asian & Other	1.17 (1.11–1.23)	6.454 × 10^−10^	1.05 (1–1.1)	0.035
Not US Born (Ref: US Born)	1 (0.95–1.05)	0.852	1.45 (1.39–1.52)	4.104 × 10^−62^
Socioeconomics				
Not Employed (Ref: Employed)	0.98 (0.94–1.01)	0.130	0.58 (0.56–0.59)	1.369 × 10^−307^
Education (Ref: ≥Bachelor's degree)				
<High school	1.71 (1.65–1.76)	2.078 × 10^−221^	1.09 (1.06–1.12)	2.240 × 10^−08^
Some college	2.17 (2.07–2.27)	6.891 × 10^−257^	0.93 (0.89–0.97)	7.224 × 10^−04^
Income (Ref: 150k more)				
<35,000	3.79 (3.58–4.01)	<.000	3.83 (3.65–4.02)	<.000
35,000–74,999	2.43 (2.31–2.55)	7.200 × 10^−264^	3.96 (3.8–4.12)	<.000
75,000–149,999	1.49 (1.42–1.56)	1.002 × 10^−58^	2.31 (2.23–2.4)	<.000
Marital status (Ref: married)				
Never married	0.84 (0.81–0.88)	4.425 × 10^−15^	0.88 (0.85–0.91)	6.018 × 10^−11^
Other	1 (0.96–1.04)	0.845	0.91 (0.88–0.95)	3.355 × 10^−07^
Insurance				
No Insurance Coverage (Ref: any)	1.67 (1.52–1.84)	2.156 × 10^−26^	2.37 (2.12–2.65)	2.596 × 10^−52^
Insurance not accepted (Ref: accepted)	0.97 (0.93–1.01)	0.176	1.9 (1.83–1.97)	9.357 × 10^−235^
No Medical visit (12 mo.) (Ref: any)	1.44 (1.38–1.51)	1.866 × 10^−58^	1.06 (1.01–1.1)	8.183 × 10^−03^
Housing				
Home (Ref: Owned)				
Rent or Other	1.38 (1.34–1.44)	1.450 × 10^−68^	1.08 (1.05–1.12)	3.694 × 10^−06^
How Long Live Here (Ref: 11+ years)				
<=2 years	1.24 (1.19–1.29)	1.359 × 10^−25^	1.05 (1.01–1.09)	6.880 × 10^−03^
3–10 years	1.18 (1.14–1.22)	1.960 × 10^−19^	1.06 (1.03–1.1)	8.094 × 10^−05^
Stability concern (Ref: no concern)	1.38 (1.32–1.44)	2.580 × 10^−47^	2.21 (2.11–2.32)	5.409 × 10^−241^

Ref., reference group; mo., months.

**Figure 1:**
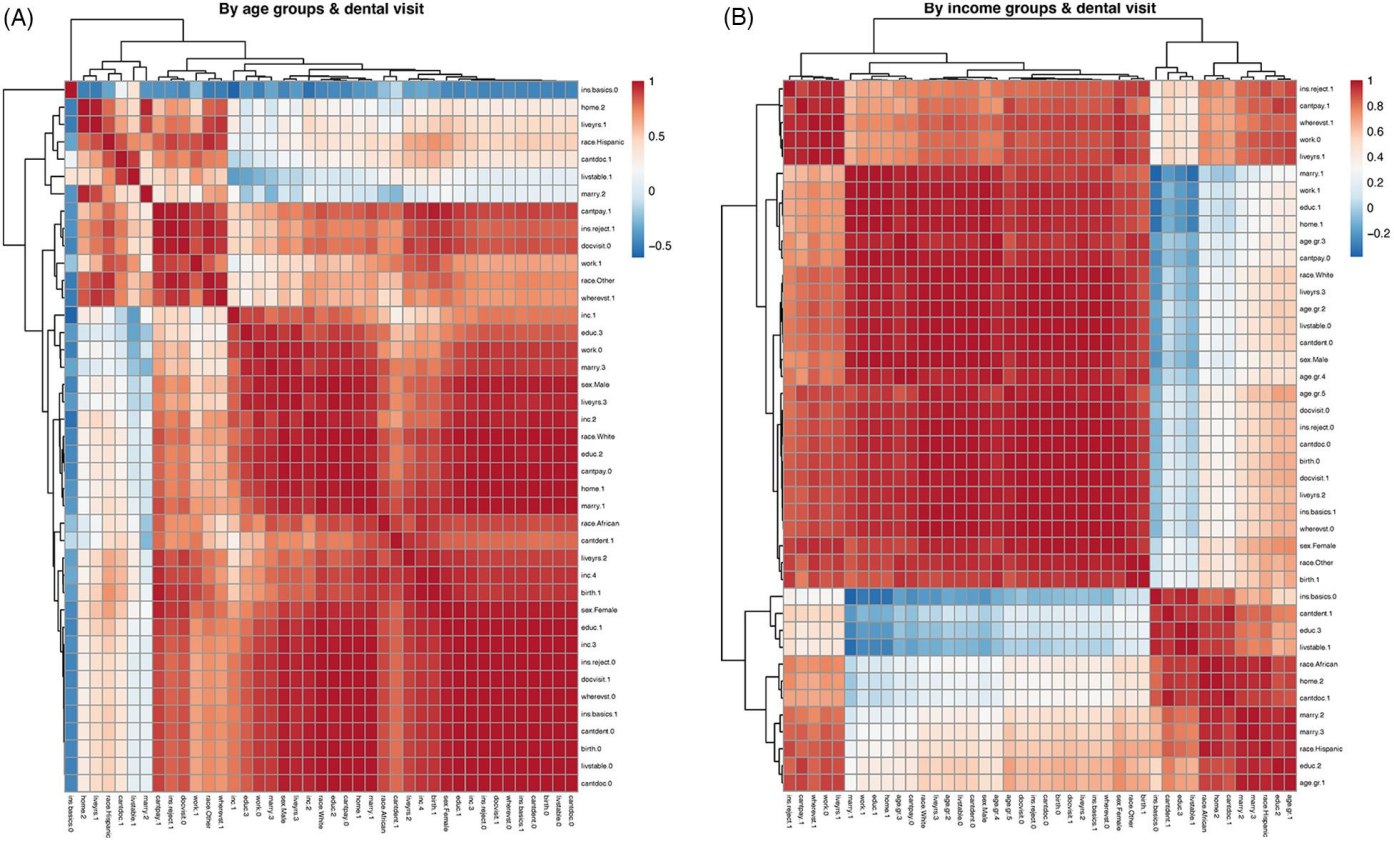
Correlations of social determinants by age or income group vs dental visit status. **(A,B)** Pearson correlation heatmaps of social determinants of health, stratified by age **(A)** and income group **(B)**, among participants with or without oral health care utilization. These exploratory visualizations illustrate clustering patterns of social vulnerability and do not represent causal or predictive modeling. The color bar indicates the strength of Pearson correlation coefficients. Dendrograms indicate the closeness of social determinants, which were sorted into block structures. See the Result section for descriptions of closely correlated clusters of social determinant factors. See Supplementary Figure S1 and the accompanying Box for details on data labeling annotations.

To complement the regression models, exploratory hierarchical clustering analyses were performed using Pearson correlation coefficients and Euclidean distance matrices. This unsupervised approach was used to visualize the patterns and proximity of social determinants among participants stratified by age group and income level in relation to oral health care utilization (Figures 1A,B). The goal was not causal inference, but to identify recurring clusters of vulnerability (e.g., low income, housing instability, lack of insurance) that may co-occur among those who did not receive oral health care. The “pheatmap” function of the R “pheatmap” package was used for Figures 1A,B.

## Results

### Characteristics of analyzed *All of US* participants based on their age groups

A total of 127,886 *All of Us* participants were included in the analyses. In demographics, participants aged 66 years and older comprised a higher proportion of the study population (35%). Female participants also overrepresented the study population, particularly in younger age groups. The majority of participants were non-Hispanic White (74.3%), with an increasing proportion as age increases. There are about 10% of participants who were not born in the US. For the socioeconomic factors, there are different trends for housing instability, marital status and income levels as the age increases. The percentage of participants with an annual income of 34,999 or less (the lowest group) decreases as age increases. Older participants are more likely to own their homes and less likely to have housing stability concerns. The youngest participants, aged 18–35 years, were less likely to be married and had lower educational levels (high school or less, 12.7%, some college, 27.3%) (Table 1).

### Characteristics of analyzed *All of Us* participants based on their income groups

Across income groups, the percentage of male and non-Hispanic white participants increases as age increases. High income earners (income 150,000 or more) are more likely to be married (86.8%), employed (71.2%), have higher education (88.9% with bachelor's degree or more), and owned their home (88.0%). They also reported less housing concerns (1.3%) (Table 3).

### Insurance and health care access

**Figure 2:**
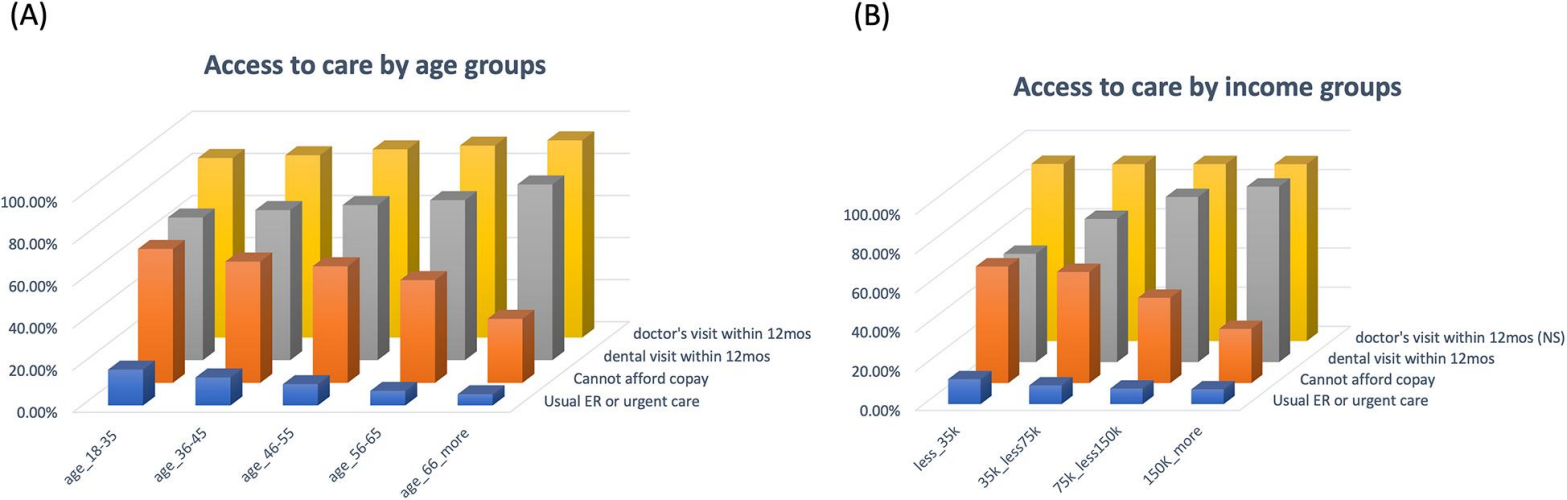
Access to care by age and income groups. **(A)** The distribution of percentages of participants who visited a medical doctor or a dentist within the past 12 months, were worried about paying the copay, or who commonly used the ER or urgent care across age groups. **(B)** The distribution of percentages of participants who visited a medical doctor or a dentist within the past 12 months, were worried about paying the copay, or commonly used ER or urgent care across the income groups.

For health care access and utilization, both the prevalence of medical visits and dental visits increase as age increases. The youngest age group reported the highest rate of insurance not accepted at the doctor's office (19.3%), cannot afford copay (63.6%), and are more likely to use emergency or urgent care services (17.0%). The youngest age group also reported the lowest prevalence of visiting a doctor's office (85.3%) or a dental office (67.8%) within the past 12 months (Figure 2) (Tables 1, 3).

Across the income groups, there were no significant differences for participants' medical visit within the past 12 months, but a drastic variation for the dental visit within the past 12 months (55.2%, 73%, 84.2%, 89.5%, across the income groups, lowest to the highest, respectively). Participants who had annual income lower than 35,000 were more likely to use emergency or urgent care services (12.7%), reported higher rate of insurance not accepted (18.8%), and much more worried about copay affordability (59.5%).

### Multivariate logistic regression results for oral health care utilization and perceived copay affordability

A multivariate logistic regression was conducted to examine the adjusted odds ratio (OR) for receiving no oral health care within the past 12 months, based on various social determinants. Compared to their respective reference groups, participants who were younger, male, of a race other than non-Hispanic white, had lower educational attainment, lower income, lacked insurance coverage, were married, or had not seen a doctor within the past year had significantly higher odds of not receiving oral health care in the past 12 months. Similarly, renters, those who had lived at their current residence for 10 years or less, and those who reported housing instability concerns also had higher odds of not having received recent oral health care (Table 4).

The associations between social determinants and the inability to pay for health services were also examined. Two separate logistic regression models were conducted using the same set of covariates to examine their associations with 1. not receiving oral health care within the past 12 months, and 2. worry about affording copay. This approach allows us to observe whether similar social determinants are independently associated with both outcomes. Younger age, lower educational and income levels, lack of insurance, renting status, and concerns about housing stability were all significantly associated with a higher likelihood of being unable to afford health services. In contrast to the factors associated with dental visit status, being born in the United States, not being married, and having insurance accepted at the doctor's office were significantly associated with a greater ability for participants to pay for copay.

### Social determinants among those without dental care across age groups

**Figure 3:**
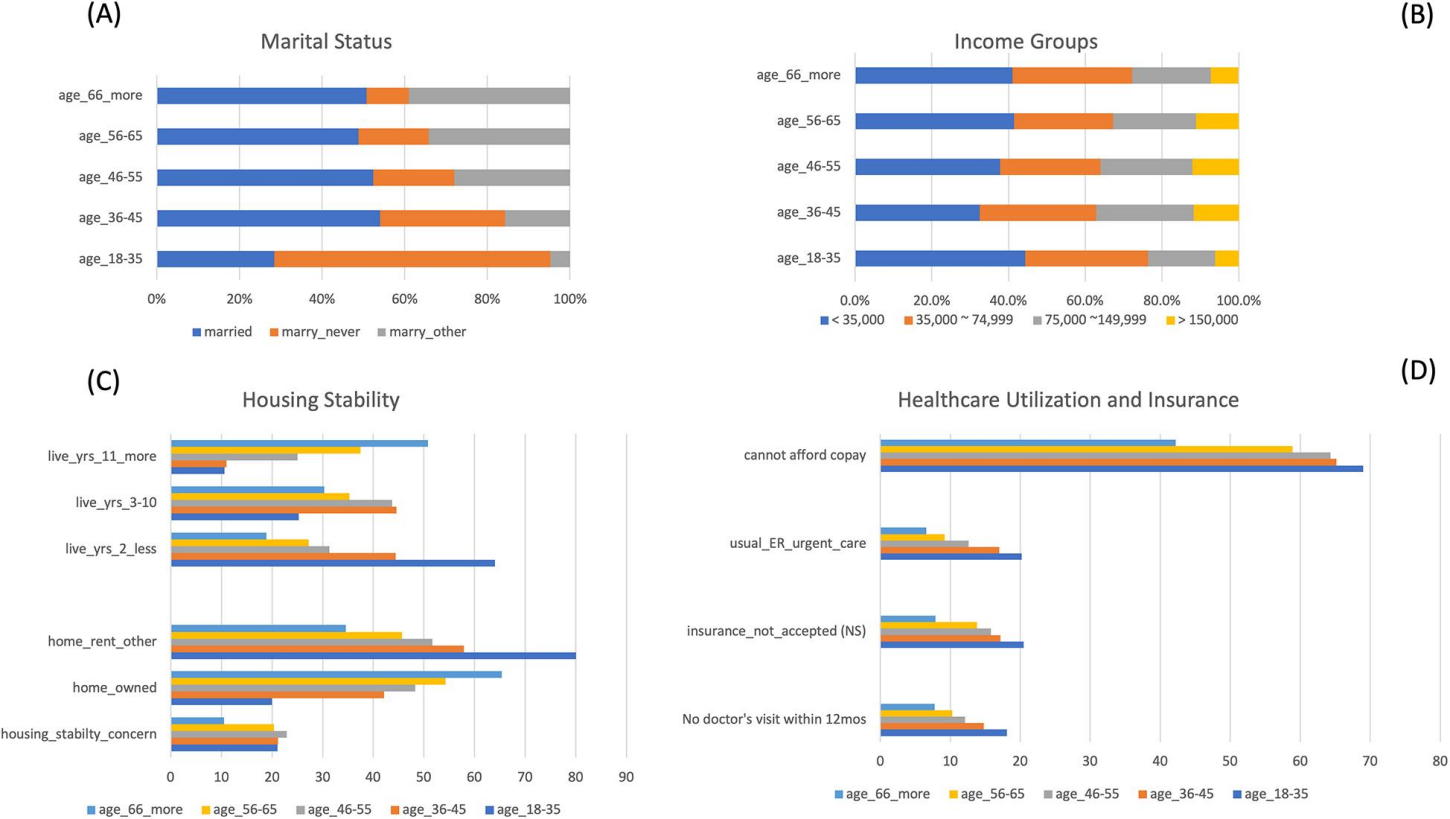
Significant social determinants among participants who did not see a dentist within the past 12 months, by age group. **(A)** Among participants who did not visit a dentist within the past 12 months, those in the youngest age group were the least likely to be married and more likely to have never been married. **(B)** Among participants who did not visit a dentist within the past 12 months, those in the youngest and the oldest age groups had the highest percentages in the lowest income group and the lowest percentages in the highest income group. **(C)** Among participants who did not visit a dentist within the past 12 months, those in the youngest age group were the least likely to own a home and were more likely to have lived in their current residence for 2 years or less. **(D)** Among participants who did not visit a dentist within the past 12 months, those in the youngest age group had the least access to care, with the highest percentage reporting they could not afford the copay. They were also more likely to use the ER or urgent care, report that their insurance was not accepted by a doctor's office, and to have not seen a medical doctor in the past 12 months.

Stratified analyses were conducted by age groups and the participants' dental visit status. Detail output for each age group is provided in Supplementary Table S1. We presented in Figure 3 that among the youngest participants (age 18–35 years old), they were more likely to never be married (66.9%), had the lowest income (<35,000, 43%), rented or other housing arrangements (80%), and lived 2 years or less in their current living places (64%). They also reported the highest rate of not seeing a doctor over the past 12 months (18.1%), insurance not accepted (20.5%), worry about copay (69%), and more commonly use emergency or urgent care services (20.2%). The housing stability concerns are consistently high among these without dental care access (21.1%, 21.2%, 22.9%, 20.4%, 10.5%, youngest to the oldest age group, respectively), comparing to the mean of 9.7% in the total analyzed samples (Figure 3).

### Correlations of social determinants by age groups and dental visit

Unlike the regression model, which assesses the independent association of each variable with oral health care access, the clustering analysis in Figure 1 was used to examine the relational patterns among social determinants. These exploratory heatmaps revealed groupings of correlated disadvantages—such as lack of insurance, unstable housing, and lower education—among participants without oral health care utilization. Based on data stratified by age groups and dental visit status (Supplementary Table S1), we observed hierarchies of correlations among social determinants of health, including insurance status, income, employment, marital status, race, and housing stability. Lack of any insurance coverage (labeled as ins.basic.0 in Figure 1A) was negatively correlated with most social determinants examined, except for concerns about housing stability (labeled as livstable.1). Participants who reported an inability to afford healthcare visits (cantpay.1) were also more likely to report that their health insurance was not accepted (ins.reject.1). These participants were less likely to have had a doctor's visit within the past 12 months (docvisit.0). Details of the data labeling process are provided in Supplementary Figure S1 and the accompanying Box (Figure 1A).

### Correlations of social determinants by income groups and dental visit

We conducted similar correlation analyses using data stratified by income groups and dental visit status (Supplementary Table S2). As in Figure 1B, we observed a similar hierarchical clustering of social determinants: participants without any health insurance coverage (labeled as ins.basic.0) reported more financial constraints to visiting a dentist (labeled as cantdent.1), lower educational attainment (high school or less, labeled as educ.3), and greater concerns about housing stability (labeled as livstable.1). Another cluster is among those whose insurances not accepted at doctor's office (labeled as ins.reject.1), not employed (labeled as work.0), worried about the copay (labeled as cantpay.1) and lived 2 years or less in their current living places (labeled as liveyrs.1). Details of the data labeling annotations are provided in Supplementary Figure S1 and the accompanying Box (Figure 1B).

### Self-reported difficulties and health conditions by age groups

**Figure 4:**
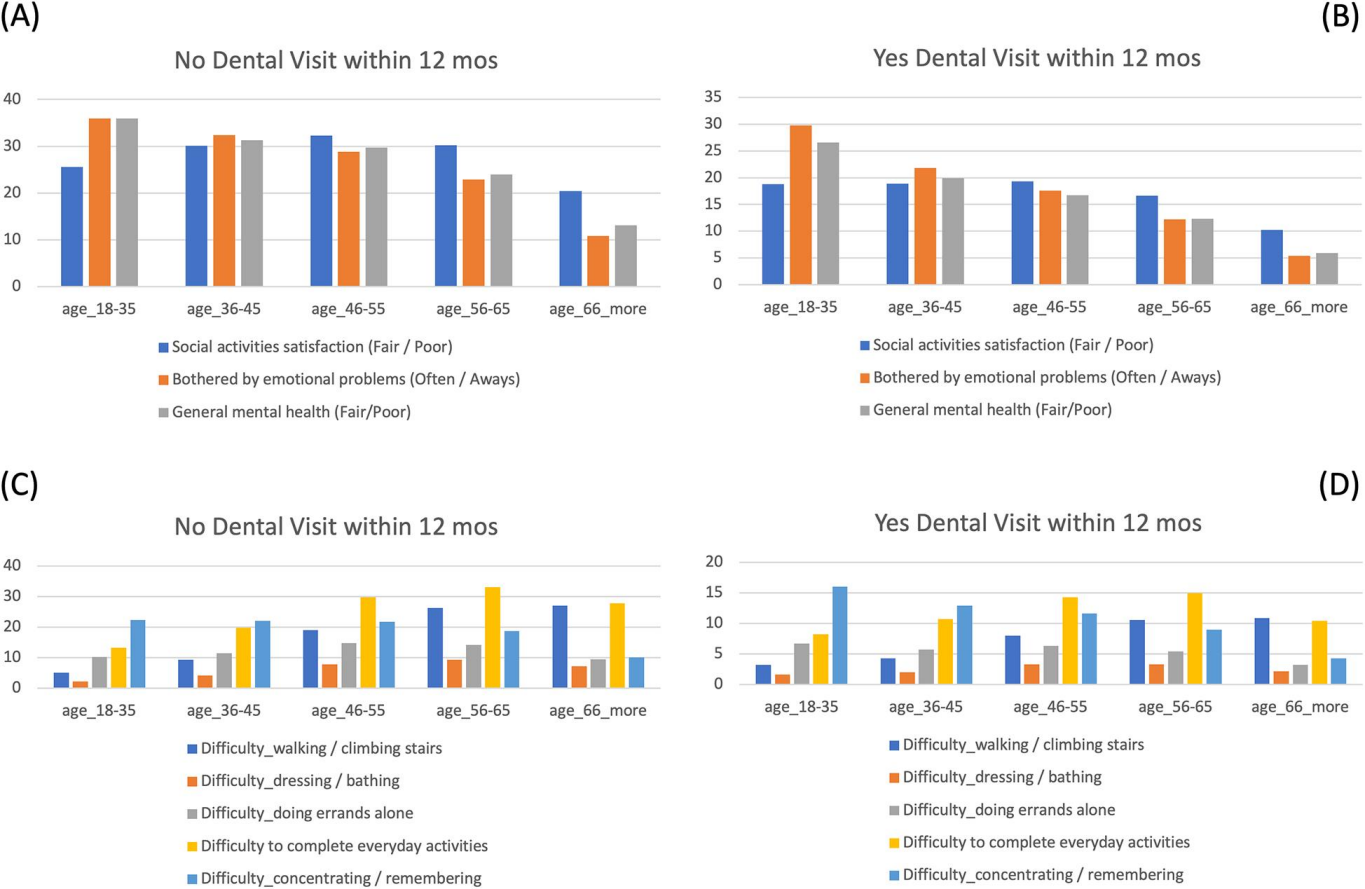
Comparisons of self-reported mental health and physical difficulties by dental care access across age groups. **(A,B)** Percentages of participants who reported worse mental health conditions and did not visit a dentist within the past 12 months across age groups **(A)**, compared to those who did visit a dentist **(B)** Their prevalence of worse mental health conditions was much higher among those without dental care access, especially in the younger age group. **(C,D)**. Percentages of participants who reported physical difficulties and did not visit a dentist within the past 12 months across age groups **(C)**, compared to those who did visit a dentist **(D)** The prevalence of physical function limitation was much higher among those without dental care access, especially in the older age groups.

As shown in Table 2, physical difficulty with walking and climbing stairs increased with age, with reported rates of 3.8%, 5.7%, 10.9%, 14.3%, and 13.6%, from the youngest to the oldest group, respectively. In contrast, participants aged 18–35 years old were more likely to report cognitive difficulties such as problems with concentrating, remembering, or decision making (18% vs. overall mean of 11%), as well as higher rates of fair/poor mental health (29.6% vs. mean 16%), and more likely to be bothered by emotional problems (often/always, 31.8% vs. mean 17%) (Figure 4, Table 2, Supplementary Table S3).

Self-reported health and functional difficulties were further examined by age groups and recent dental visit status (Supplementary Table S3 and Figure 4). These comparisons were not modeled as outcomes but are presented descriptively to illustrate how psychosocial and physical challenges vary across age groups and appear more pronounced among individuals without recent oral health care utilization. Across all age groups, participants who had not visited a dentist in the past 12 months reported significantly worse social satisfaction, poorer mental health, and were more likely to experience emotional distress. In terms of physical limitations, they also reported greater difficulty walking, climbing stairs, doing errands alone, and completing everyday activities. These differences were significant across all age groups, though most prominent among older participants.

These findings are intended to contextualize the broader landscape of unmet needs and vulnerabilities across the lifespan. Stratified comparisons by age and utilization status were used to illustrate how psychosocial and functional challenges differ by life stages and are more common in those lacking recent oral health care.

## Discussion

This study provides a comprehensive, stratified analysis of significant social determinants affecting dental care access across the lifespan. Our results demonstrated that lack of access to oral health care correlates with various social factors differently across age groups, particularly the youngest and oldest—both vulnerable in different ways. Importantly, by incorporating insurance status, affordability, housing, marital status, social satisfaction, mental health, and self-reported physical limitations, we identify novel distinct and overlapping barriers to dental care that vary by age and socioeconomic context. It is important to note that while this study refers to access, the operational measure is based on actual utilization. Although not equivalent, dental visit behavior is widely accepted as a practical and meaningful proxy for access in population-level analyses where information on unmet need is not available. The strength of this study is leverage on the large sample size data from the NIH *All of Us* research program and the comprehensive, multi-facet approach to understanding of factors associated with dental care access. Prior research in the social ecological model (16) and the WHO SDOH framework (17) proposed a comprehensive framework to investigate the complex problem of health equity. This report considers more diverse social determinants in the general population across different age groups. The presented findings highlighted an under detected vulnerable group in young adulthood (age 18–35 years old) that has slightly different SDOH profiles than the rest of the population. Given our rapidly aging population structure, the desperate need for this younger age group who will be our prime source for societal productivity should not be ignored.

Consistent with previous research, income emerged as a foundational determinant of dental care utilization. Individuals in the highest income group (annual household income 150,000 or more) were nearly twice as likely to have had a dental visit in the past 12 months compared to those in the lowest (less than 35,000), with this gradient persisting across all age groups (6, 20). Education and homeownership—markers of social and financial stability—also demonstrated strong positive associations with utilization, aligning with broader findings that dental care is often contingent on structural privilege (21, 22). Racial and ethnic disparities remained persistent even after adjusting for social and economic factors. White participants had higher rates of dental utilization across all age groups, while Black and Hispanic individuals faced compounded disadvantages—lower insurance coverage, higher cost barriers, and less homeownership. These findings align with prior work documenting structural disadvantage in oral health access and point to the need for policy and provider-level reforms that address equitable access, and provider distribution (23, 24).

Notably, this study highlights that functional disability may be an important, yet under-recognized factor influencing access to dental care, as suggested by its higher prevalence among those without recent utilization. Participants reporting difficulty concentrating, walking, bathing, or running errands were more likely to forgo dental care, with these associations particularly pronounced among older adults. This pattern points to barriers such as transportation, physical accessibility, and system navigation challenges, underscoring the need for home-based or mobile dental care programs (25). Across all age groups, individuals who had not seen a dentist most frequently cited financial constraints and insurance rejection as primary barriers. Among younger adults aged 18–35, cost was the most commonly reported reason for non-utilization, despite this group reporting the fewest physical limitations. This finding reinforces the persistent policy gap in dental coverage, especially for those not covered by employer-based insurance or public programs (26). Our findings lend support ongoing calls for the inclusion of dental benefits in Medicare, Medicaid, and emerging universal health coverage frameworks (27).

The age-based stratification also revealed important generational contrasts. While older adults benefited from higher insurance coverage and homeownership rates, they also reported more functional impairment and lower educational attainment. Conversely, younger adults were more likely to be uninsured, renters, and racially diverse, reported more insurance not accepted, less physical limitations, and worse mental health with more emotional problems. These contrasting profiles suggest that interventions must be tailored not only to income but to life stage and cumulative disadvantage (23, 24). Although self-reported functional and psychosocial health indicators were not modeled as outcomes in this study, descriptive analyses revealed distinct life stage disparities: younger adults were more likely to report emotional and mental health difficulties, while older adults more often experienced physical limitations. These patterns were exacerbated in those who did not receive oral health care. Future studies could explore these associations more formally using multivariate modeling frameworks.

## Limitations

This study has several limitations. Due to the cross-sectional and correlational study design, the findings reflect associations only, and causal interpretations cannot be inferred. Although the sample size is substantial, detailed geographic and insurance information was not included in this analysis due to concerns about further stratification and multiple testing. The analytical sample (*n* = 127,886), representing approximately 20% of participants who completed the entry “Basics” survey, is not representative of the general U.S. population. Participants retained in the analytical sample (i.e., those with complete data on outcome variables and covariates) were older, less racially diverse, more likely to be females, and had generally higher socioeconomic status along with fewer access barriers. As a result, our estimates of access inequities in this report may be conservative.

Additionally, all data analyzed were based on self-reported survey responses, which may be subject to recall or misclassification bias. In particular, the variable reflecting concern about copay affordability captures a subjective perception of financial strain rather than an objective measure of forgone care or service denial. However, such perceptions are themselves meaningful early-stage barriers, especially among younger or lower-income populations, as they may deter individuals from even attempting to seek care. Also, self-report biases typically attenuate observed associations, biasing results toward the null. Finally, although the *All of Us* dataset allows for longitudinal follow-up, only baseline cross-sectional data were used in the present analyses.

## Future recommendations

The present findings highlight the need for a multi-layered policy response. This includes expanding public dental insurance, reducing administrative barriers to care, supporting mobile and community-based services, and integrating oral health equity goals into public health surveillance and primary care frameworks (28). A thoughtfully designed dental benefit should begin with preventive and restorative services in early adulthood and include behavior-based incentives to encourage utilization and engagement. States can incorporate these benefits into managed care organizations and align them with value-based care goals –exemplified by Iowa's Health and Wellness Plan, which offers free Medicaid coverage to low-income adults aged 19–64 in the first year. To retain no-cost coverage, enrollees must complete a health risk assessment and an annual wellness or dental exam through the Healthy Behaviors Program. While the program expands preventive care access, the wellness requirements may pose barriers for individuals with limited provider access or transportation.

A promising strategy is to expand partnerships with Dental Support Organizations (DSOs) and Federally Qualified Health Centers (FQHCs), while also engaging non-traditional networks such as mobile clinics, school-based dental programs, and teledentistry platforms. States can further incentivize provider participation not only through increased reimbursement but also via tax credits or public recognition for delivering philanthropic dental services to Medicaid or uninsured populations. These “community benefit” credits could be integrated into state licensing renewal or Medicaid contracting criteria.

To ensure sustainability and system-wide effectiveness, a key operational opportunity lies in leveraging integrated electronic health record (EHR) systems —such as Epic Systems—used by many DSOs and health systems. When medical and dental services share a unified EHR, providers can streamline referrals, coordinate chronic disease management, and access complete patient histories. Systems like Epic also support structured data export for research, quality improvement, and cross-sector evaluation—tracking outcomes such as ER diversion, improved hemoglobin A1c control, or increased uptake of preventive services. By aligning policy design with clinical informatics and interprofessional collaboration, dental benefits can be sustainably embedded into public insurance programs, advancing both equity and 5faith-based health systems and religiously affiliated organizations offer mission-driven models of integrated, charitable dental care, making them ideal partners in expanding public dental benefits. Examples include AdventHealth, which emphasizes whole-person care and operates community dental services; Remote Area Medical, which delivers free pop-up clinics across the U.S.; and Christian Community Health Fellowship, a network of providers that runs dental clinics within FQHCs and nonprofit centers. These organizations provide more than infrastructure—they offer trusted, community-rooted care models that align naturally with public benefit goals. With targeted support such as tax incentives, licensing recognition for charitable care, or grant-funded Epic integration, these faith-based entities could serve as anchor institutions for sustainable, value-based medical-dental care delivery. Engaging them not only broadens the service network but also strengthens community ties, promotes health equity, and supports state in delivering integrated oral health through public insurance programs.

Lastly, the *All of Us* dataset—along with large biobank resources such as the VA Million Veteran Program (29)—provides a uniquely inclusive platform to study these dynamics. Future longitudinal analyses can investigate causal relationships to assess how poor dental access may drive or exacerbate declines in physical and mental health over time. Studies focused on young adults— especially those experiencing housing instability, low income, or multiple minority identities—could help clarify why mental health burdens are more prevalent among those without dental access.

## Data Availability

Publicly available datasets were analyzed in this study. This data can be found here: https://www.researchallofus.org/data-tools/workbench/.
