# Supplementary material for: Access to oral health care and its social determinants across the lifespan in the United States
Source: Front Oral Health. 2025 Sep 11;6:1619983. doi: 10.3389/froh.2025.1619983 (PMC12460411; doi:10.3389/froh.2025.1619983)
Supplement: Supplementary file 2 [file Datasheet1.pdf]

Supplementary Figure S1. Flow chart of analytical sample selection using AoU CDR v8. *Accessed on 4/22/2025*

Person who completed health care access & utilization (original n=305,860)
(with non-missing values of age, gender, race)
N = 297,959 to start merging and filtering to the final sample size

Dataset / Covariates	No. participants with missing values	% of N participants with missing values
Basics (original n=628,651, merged)		
Education (educ.1=BS+, 3 levels)	3916	1.31%
Employment (work.1=Yes)	4079	1.37%
Income (inc.1=35k less, 4 levels)	36178	12.14%
Having any insurance (ins.basics.1=Yes)	4044	1.36%
Home ownership (home.1=own)	5843	1.96%
How long live here (liveyrs.1=2 yrs less, 3 levels)	1957	0.66%
Stable housing concern (livstable.1=Yes)	1848	0.62%
Marital status (marry.1=yes, 2=never, 3=other)	23645	7.94%
Birthplace (birth.0=US born)	1270	0.43%
Difficulties in walking / climbing	51036	17.1%
Difficulties in dressing / bathing	50852	17.1%
Difficulties in doing errands	50835	17.1%
Difficulties in concentrating / memorizing	52668	17.7%
Heath Care Access & Utilization (out of the N=297,959)		
Insurance not accepted (ins.reject.1=not accepted)	6053	2.03%
Visit a medical doctor within 12 months (docvisit.1=yes)	11623	7.59%
Visit a dentist within 12 months	58696	19.7%
Usual place for care (doctor's office, ER/urgent) (wherevisit.0=doctor's office)	18847	6.33%
Cannot afford copayment (cantpay.1=worry about)	9469	3.18%
Cannot afford to see a doctor (cantdoc.1=yes)	31302	10.5%
Cannot afford to see a dentist (cantdent.1=yes)	21831	7.33%
Overall Health (original n = 577,969, merged)		
General mental health	13435	4.51%
Social activities satisfaction	1987	0.67%
Emotional problem over past 7 days	1989	0.67%
Ability to complete everyday activities	1679	0.56%

➔ after removing any participants with missing values ➔ **final sample** = 127,886

Box. Characteristics among *All of Us* participants who completed different questionnaires using AoU CDR v8. *Accessed on 4/22/2025 (analytical) and 7/16/2025 (data browser)*

**Questionnaires**	**Basics**	**Overall Health**	**Health Care Access & Utilization**	**Analytical**
Total (data browser)	633,540	582,680	305,860	127,886
Age				
mean (SD)	55.37 (16.9)	55.61 (16.9)	56.73 (16.9)	56.39 (16.2)
18-35 (agegr.1)	100081 (15.8)	90813 (15.6)	43088 (14.1)	16832 (13.2)
36-45 (agegr.2)	102935 (16.2)	93066 (16.0)	45011 (14.7)	20344 (15.9)
46-55 (agegr.3)	97827 (15.4)	88722 (15.2)	43693 (14.3)	20532 (16.0)
56-65 (agegr.4)	122675 (19.4)	113355 (19.5)	56306 (18.4)	25279 (19.8)
66+ (agegr.5)	205146 (32.4)	192013 (33.0)	109861 (35.9)	44899 (35.1)
Biological Sex*				
Male (sex.Male)	228652 (36.1)	213579 (36.7)	103335 (33.7)	42997 (33.6)
Race/Ethnicity*				
White (race.White)	343091 (54.2)	312799 (53.7)	201727 (65.9)	95034 (74.3)
Black (race.African)	97508 (15.4)	92257 (15.8)	27733 (9.1)	8637 (6.8)
Hispanic (race.Hispanic)	112461 (17.8)	105017 (18.0)	40782 (13.3)	12965 (10.1)
Asian & Other (race.other)	64687 (10.2)	58015 (10.0)	27717 (9.1)	11250 (8.8)
Insurance Status*				
Yes(any) (ins.basic.1=yes)	578040 (91.2)	527246 (90.5)	283089 (92.6)	125834 (98.4)
Income Levels				
<34,999 (inc.1)	191540 (30.2)	175161 (30.1)	67968 (22.2)	26524 (20.7)
35,000-74,999 (inc.2)	125840 (19.9)	112274 (19.3)	67970 (22.2)	32572 (25.5)
75,000-149,999 (inc.3)	125620 (19.8)	113585 (19.5)	77080 (25.2)	41308 (32.3)
150,000 more (inc.4)	75920 (12.0)	69505 (11.9)	48763 (15.9)	27482 (21.5)
missing	*114620 (18.1)*	*112155 (19.2)*	*44079 (14.4)*	*0*
Health Utilization*				
Medical visit yes (docvisit.1)	n/a	n/a	253880 (83.0)	115462 (90.0)
Dental visit yes	n/a	n/a	177620 (58.1)	97763 (76.5)
Cannot afford copay	n/a	n/a	144720 (47.3)	59792 (46.7)
Insurance not accepted	n/a	n/a	36400 (11.9)	15529 (12.1)
Difficulties in*				
Concentrating	55100 (8.7)	48027 (8.2)	28630 (9.4)	14320 (11.2)
Walking / climbing	59060 (9.3)	52564 (9.0)	29885 (9.8)	13745 (10.7)
Social & Emotional (fair / poor)*				
Mental health	n/a	106840 (18.3)	49077 (16.0)	20825 (16.3)
Social satisfaction	n/a	110020 (18.9)	52877 (17.3)	23701 (18.5)
Housing Stability Concern*				
Yes	104100 (16.4)	94135 (16.2)	34050 (11.1)	12341 (9.7)

** missing data % not provided here.*
